# Mitochondrial DNA-Based Identification of Forensically Important Flesh Flies (Diptera: Sarcophagidae) in Thailand

**DOI:** 10.3390/insects11010002

**Published:** 2019-12-18

**Authors:** Chutharat Samerjai, Kabkaew L. Sukontason, Narin Sontigun, Kom Sukontason, Tunwadee Klong-klaew, Theeraphap Chareonviriyaphap, Hiromu Kurahashi, Sven Klimpel, Judith Kochmann, Atiporn Saeung, Pradya Somboon, Anchalee Wannasan

**Affiliations:** 1 Center in Insect Vector Study, Department of Parasitology, Faculty of Medicine, Chiang Mai University, Chiang Mai 50200, Thailand; chutharat.smj@gmail.com (C.S.); kabkaew.s@cmu.ac.th (K.L.S.); narinsontigun@gmail.com (N.S.); kom.s@cmu.ac.th (K.S.); atiporn.s@cmu.ac.th (A.S.); pradya.somboon@cmu.ac.th (P.S.); 2 Graduate School, Chiang Mai University, Chiang Mai 50200, Thailand; 3 School of Allied Health Sciences, Walailak University, Nakhonsithammarat 80161, Thailand; somtunwa@gmail.com; 4 Department of Entomology, Faculty of Agriculture, Kasetsart University, Bangkok 10900, Thailand; faasthc@ku.ac.th; 5 Department of Medical Entomology, National Institute of Infectious Disease, Toyama 1-23-1, Shinjuku-ku, Tokyo 162-8640, Japan; MLB15110@nifty.com; 6 Institute for Ecology, Evolution and Diversity, Faculty of Biological Sciences, Goethe University, Max-von-Laue-Str.13; Senckenberg Biodiversity and Climate Research Centre (SbiK-F), 60438 Frankfurt am Main, Germany; Klimpel@bio.uni-frankfurt.de (S.K.); judith.kochmann@senckenberg.de (J.K.)

**Keywords:** forensic entomology, molecular identification, *COI*, *COII*, flesh flies, Thailand

## Abstract

Flesh flies (Sarcophagidae) are necrophagous insects initially colonizing on a corpse. The species-specific developmental data of the flies collected from a death scene can be used to estimate the minimum postmortem interval (PMI_min_). Thus, the first crucial step is to correctly identify the fly species. Because of the high similarity among species of flesh flies, DNA-based identification is considered more favorable than morphology-based identification. In this study, we demonstrated the effectiveness of combined sequences (2216 to 2218 bp) of cytochrome c oxidase subunit I and II genes (*COI* and *COII*) for identification of the following 14 forensically important flesh fly species in Thailand: *Boettcherisca nathani* Lopes, *Fengia ostindicae* (Senior-White), *Harpagophalla kempi* (Senior-White), *Liopygia ruficornis* (Fabricius), *Lioproctia pattoni* (Senior-White), *Lioproctia saprianovae* (Pape & Bänziger), *Parasarcophaga albiceps* (Meigen), *Parasarcophaga brevicornis* (Ho), *Parasarcophaga dux* (Thomson), *Parasarcophaga misera* (Walker), *Sarcorohdendorfia antilope* (Böttcher), *Sarcorohdendorfia inextricata* (Walker), *Sarcorohdendorfia seniorwhitei* (Ho) and *Seniorwhitea princeps* (Wiedemann). Nucleotide variations of Thai flesh flies were evenly distributed throughout the *COI-COII* genes. Mean intra- and interspecific variations ranged from 0.00 to 0.96% and 5.22% to 12.31%, respectively. Using Best Match (BM) and Best Close Match (BCM) criteria, identification success for the combined genes was 100%, while the All Species Barcodes (ASB) criterion showed 76.74% success. Maximum Likelihood (ML) and Bayesian Inference (BI) phylogenetic analyses yielded similar tree topologies of monophyletic clades between species with very strong support values. The achieved sequences covering 14 forensically important flesh fly species including newly submitted sequences for *B*. *nathani*, *F*. *ostindicae* and *S*. *seniorwhitei*, can serve as a reliable reference database for further forensic entomological research in Thailand and in other areas where those species occur.

## 1. Introduction

Besides the Calliphoridae, Sarcophagidae (flesh flies) contain some of the most important carrion-breeding flies which colonize a human cadaver during the initial stages of decomposition [1]. In forensic investigations, the sarcophagids provide more precise PMI_min_ estimation than calliphorids because they are larviparous and deposit larvae directly on the cadaver and feed immediately [2]. Substantial entomological evidence has been presented for flesh flies, for example, *Bercaea africa* (Wiedemann) in Italy [3], *Liopygia ruficornis* (Fabricius) in Thailand [4] and Kuwait [5], *B. africa*, *Parasarcophaga dux* (Thomson), *Liopygia argyrostoma* (Robineau-Desvoidy), *Robineauella scoparia* (Pandelle)*, Parasarcophaga similis* (Meade) in Switzerland [6], *Seniorwhitea princeps* (Wiedemann) in Malaysia [7], and *L. argyrostoma*, *B. africa*, *Heteronychia fertoni* (Villeneuve), *Boettcherisca peregrine* (Robineau-Desvoidy) in Iran [8,9].

Among 2510 known species in 173 genera of Sarcophagidae described worldwide [10], 86 species in 31 genera have been recorded in Thailand [11]. Most adults in the subfamily Sarcophaginae share some common morphological characteristics which include grey-black longitudinal stripes on the thorax, a checkerboard abdomen, and a strongly bristled body [12]. Morphological characteristics of immature and adult stages among flesh fly species are very similar, thus making identification difficult, particularly for non-expert taxonomists [13]. Since the developmental times of flesh flies are species specific, the correct identification at the species level is a primary step for estimating the PMI_min_ [14,15]. Therefore, a potential tool is needed which can discriminate the species regardless of life-history stage is needed [16].

Recently, DNA-based identification which requires only a small sample of any life stage, has been extensively used and has become a reliable routine tool in forensic entomology [17,18,19]. Among applied genetic markers, mitochondrial cytochrome c oxidase subunit I (*COI*) has been widely used as a species identifier because of its beneficial features for evolutionary genetics studies, such as a high copy number per cell, a high mutation rate, and haploid maternal inheritance [20,21]. Many studies have documented the robustness of *COI* as the DNA barcode for fly species discrimination [16,17,22]. Nervertheless, the usage of short fragments or even the entire sequence of *COI* is sometimes limited in resolving phylogenetic relationships and identifying cryptic species [16,23] or species complexes [24] of some flesh flies. Several investigations suggested that using *COI* alone, as a species identifier, should be done with care and to achieve a 100% identification success, multiple markers should be used in the analyses, especially for Sarcophagidae [24,25,26].

Sequences of forensically important flesh flies have been published from different regions of the world [16,22,27,28], but they are still insufficient in the Oriental regions [2,21]. To date, a reference DNA database of forensically important flesh flies in Thailand is missing and only two genetic studies involving the *COI* and nuclear *28S* rRNA genes for only five flesh fly species have been reported [29,30]. Therefore, this study aimed to evaluate the use of combining *COI* and *COII* genes to identify 14 forensically important Thai flesh fly species and to improve the regional databases as sequence data for some species have never been reported before.

## 2. Materials and Methods 

### 2.1. Specimen Collection

**Figure 1:**
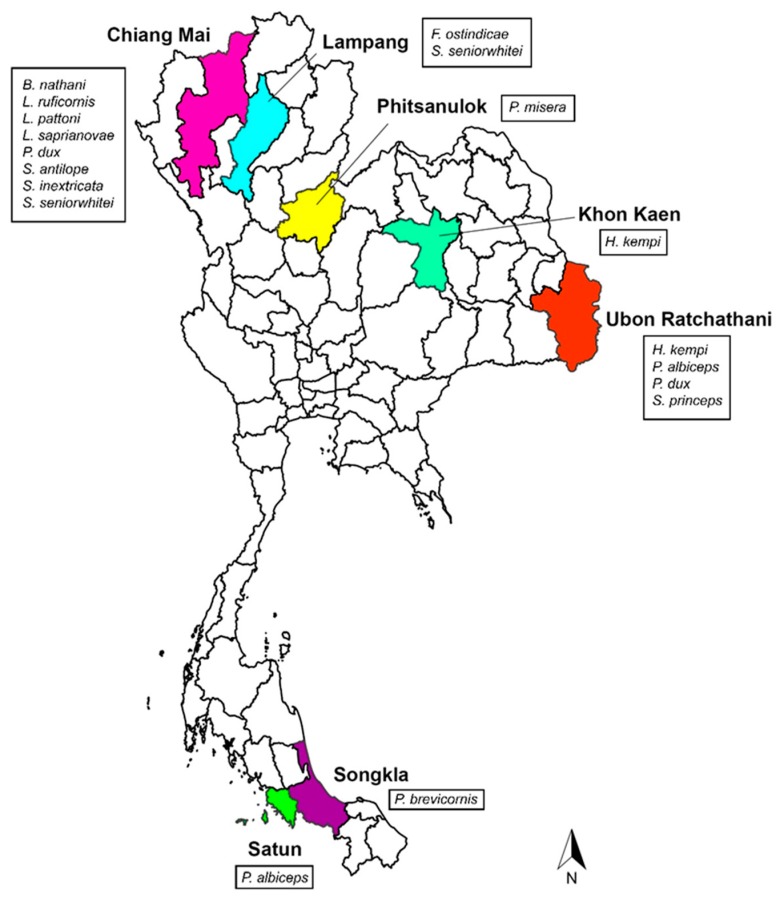
Map of Thailand showing the flesh fly species collected in 7 provinces.

From 2015 to 2016, flesh fly collections were carried out in 7 provinces of Thailand, including Chiang Mai, Lampang, Phitsanulok, Khon Kaen, Ubon Ratchathani, Songkla, and Satun (Figure 1). Collections were performed by sweeping method using 300 g of 1-day tainted beef offal as the attractive bait. After collections, specimens were frozen at −20 °C for 1 h and adult males were identified based on the comparative morphology of male genitalia, as previously described [31]. Subsequently, all identified males were preserved in 70% ethanol and kept at −20 °C until further used for molecular analysis. Additionally, different isolines of 12 flesh flies and 2 house flies (*Musca domestica* Linnaeus) from the rearing laboratory of the Department of Parasitology, Faculty of Medicine, Chiang Mai University, were included in the study. Details of collection data for all fly specimens are shown in Table 1.

### 2.2. DNA Extraction, Amplification, and Sequencing

Genomic DNA was extracted from two legs of each fly using E.Z.N.A^®^ Tissue Kit (Omega Bio-tek, Norcross, GA, USA) according to the manufacturer’s protocol. The remaining parts of the specimens were maintained as voucher specimens at the Fly Research Unit, Department of Parasitology, Faculty of Medicine, Chiang Mai University, Thailand.

For DNA amplification, two pairs of primers were used in PCR reactions. Primers of TY-J-1460 (5′-TACAATTTATCGCCTAAACTTCAGCC-3′) and C1-N-2800 (5′-CATTTCAAGCTGTGTAA-GCATC-3′) were used for generating *COI* fragments while primers C1-J-2495 (5′-CAGCTACTTT-ATGAGCTTTAGG-3′), and TK-N-3775 (5′-GAGACCATTACTTGCTTTCAGTCATCT-3′) were used for *COII* amplification [20]. Twenty-five microliters of a PCR reaction were composed of 100 ng of template DNA, 1 U of Platinum^®^ Taq DNA polymerase (Invitrogen, Carlsbad, CA, USA), 1× PCR reaction buffer (Invitrogen, Carlsbad, CA, USA), 2.5 mM MgCl_2_ (Invitrogen, Carlsbad, CA, USA), 200 µM of each dNTPs (Invitrogen, Carlsbad, CA, USA) and 0.4 µM of each primer. PCR amplifications were performed in TPersonal Combi Thermocycler (Biometra, Göttingen, Germany) consisting of a step of initial denaturation at 94 °C for 5 min, followed by 35 cycles of denaturation at 94 °C for 1 min, annealing at 50 °C (for *COI*) or 47 °C (for *COII*) for 1 min, an extension at 72 °C for 2 min, and a final extension step at 72 °C for 5 min. PCR products were electrophoresed in 1% agarose gel (Amresco, Atlanta, GA, USA), stained with RedSafe^TM^ Nucleic Acid Staining Solution (20,000×) (iNtRON Biotechnology, Inc., Seongnam, South Korea) and subsequently purified using the E.Z.N.A^®^ Cycle Pure Kit (Omega Bio-tek, Norcross, GA, USA). The purified PCR products were sent to First BASE Laboratories Sdn. Bhd. (Selangor, Malaysia) for bidirectional sequencing with the BigDye^®^ Terminator v3.1 cycle sequencing kit (Applied Biosystems, Foster City, CA, USA), using the same primers as previously used in the PCR.

### 2.3. Sequence Analysis 

The obtained *COI* and *COII* sequences from each specimen were edited manually and assembled into a combined bidirectional consensus sequence (*COI*-*COII*) using BioEdit software version 7.0.9.0. [32]. The consensus sequences were all aligned using the Clustal W algorithm implemented in MEGA 7 [33]. The genetic divergence within (intraspecific) and between (interspecific) species were calculated using the Kimura 2-parameter (K2P) model through MEGA 7 [33]. To confirm the morphological results, the sequences were individually compared with dipteran sequences in the GenBank database using the BLAST tool (available at http://blast.ncbi.nlm.nih.gov/Blast.cgi). The sequences retrieved in this study were deposited in the GenBank under the accession numbers MH765499-MH765541 (Table 1). 

### 2.4. Identification Success

Using Best Match (BM), Best Close Match (BCM) and All Species Barcodes (ASB) criteria proposed by Meier et al. [34], the percentage of correctly identified specimens of the combined *COI-COII* sequences were estimated. All parameters were performed using SpeciesIdentifier software version 1.832 [34]. 

### 2.5. Phylogenetic Analysis

To determine taxonomic relationships between the species, a phylogenetic tree was constructed using Maximum Likelihood (ML) under the GTR+I+G model in MEGA 7.0 [33], with 1000 bootstrap replications. The most appropriate model of nucleotide substitution for the *COI-COII* dataset (GTR+I+G model) was determined using jModelTest version 2.1.10 [35,36]. In addition, a Bayesian Inference analysis (BI) was conducted with MrBayes 3.2.7 [37]. Four Markov chains (three heated chains and one cold chain) were run for 200,000 generations and the trees were sampled every 100 generations. Figtree software version 1.4.4 (http://tree.bio.ed.ac.uk/software/figtree/) was used to generate the BI tree. All trees were rooted with *M*. *domestica*, whereas only branches with over 70% bootstraps were considered for phylogenetic inference [38]. 

## 3. Results

### 3.1. Sequence Analysis

The combined *COI*-*COII* sequences of all 43 specimens were successfully generated. The lengths of the fragments varied from 2216 to 2218 bp depending on the species. The identification of species by BLAST based on the sequences, was consistent with morphological identifications. For the sequence alignment, a total of 2218 aligning base positions covered three consecutive genes, i.e., *COI* (positioned 1 to 1495), tRNA-leucine (positioned 1496 to 1561), and *COII* genes (positioned 1569 to 2218). Notably, seven nucleotides of a spacer region (positioned 1562 to 1568) were found between tRNA-leucine and *COII* genes of *P. dux*, *S. princeps*, *H. kempi*, *L. ruficornis*, and *F. ostindicae*, while the remaining species showed 1 or 2 indels in the region (Table 2). Although each species showed only one pattern of nucleotide arrangement in the intergenic spacer, the pattern was not species specific. The final alignment contained 580 variable sites of which 568 sites were considered parsimony informative. The analyzed sequences showed a strong AT bias with the average nucleotide compositions of A (31.9%), T (38.1%), C (15.7%), and G (14.3%), respectively.

The tRNA-leucine gene of most examined species was conserved, except for *S*. *inextricata*, *S*. *antilope*, and *F. ostindicae*. One transition was revealed in *S. inextricata* (A↔G at position 1502) and *F. ostindicae* (C↔T at position 1531), while *S*. *antilope* showed two transitions (A↔G at position 1502, T↔C at position 1510) and one transversion (T↔G at position 1534) (Table 3). 

### 3.2. Genetic Variation

Nucleotide variation of Thai flesh flies was evenly distributed throughout the *COI*-*COII* genes. The mean intraspecific variation ranged from 0.00% to 0.96%, while the mean interspecific variation ranged from 5.22% (*P*. *albiceps*/*P*. *misera*) to 12.31% (*F*. *ostindicae*/*H*. *kempi*) (Table 4). No intraspecific variation was observed within *B*. *nathani*, *F*. *ostindicae*, *L*. *ruficornis*, *L*. *saprianovae,* and *P*. *brevicornis*. The interspecific variation within genus *Lioproctia* was 6.77% (*L*. *pattoni*/*L*. *saprianovae*), while that within genus *Parasarcophaga* was diverse from 5.22% (*P*. *albiceps*/*P*. *misera*) to 7.78% (*P*. *brevicornis*/*P*. *misera*), and within genus *Sarcorohdendorfia* varied from 6.50% (*S*. *inextricata*/*S*. *seniorwhitei*) to 6.89% (*S*. *antilope*/*S*. *seniorwhitei*).

### 3.3. Identification Success

The identification success for *COI*-*COII* sequences of flesh flies was 100% for the BM and BCM criteria. The ASB criterion presented an identification success of 76.74%. No ambiguous identifications were found for the BM and BCM criteria, whereas 23.25% ambiguous identifications were found in the ASB criterion. No incorrect identification was observed under the three criteria. The BCM and ASB criteria showed 0.00% of sequences with no matches closer than the calculated threshold (0.94%) (Table 5).

### 3.4. Phylogenetic Analyses

**Figure 2:**
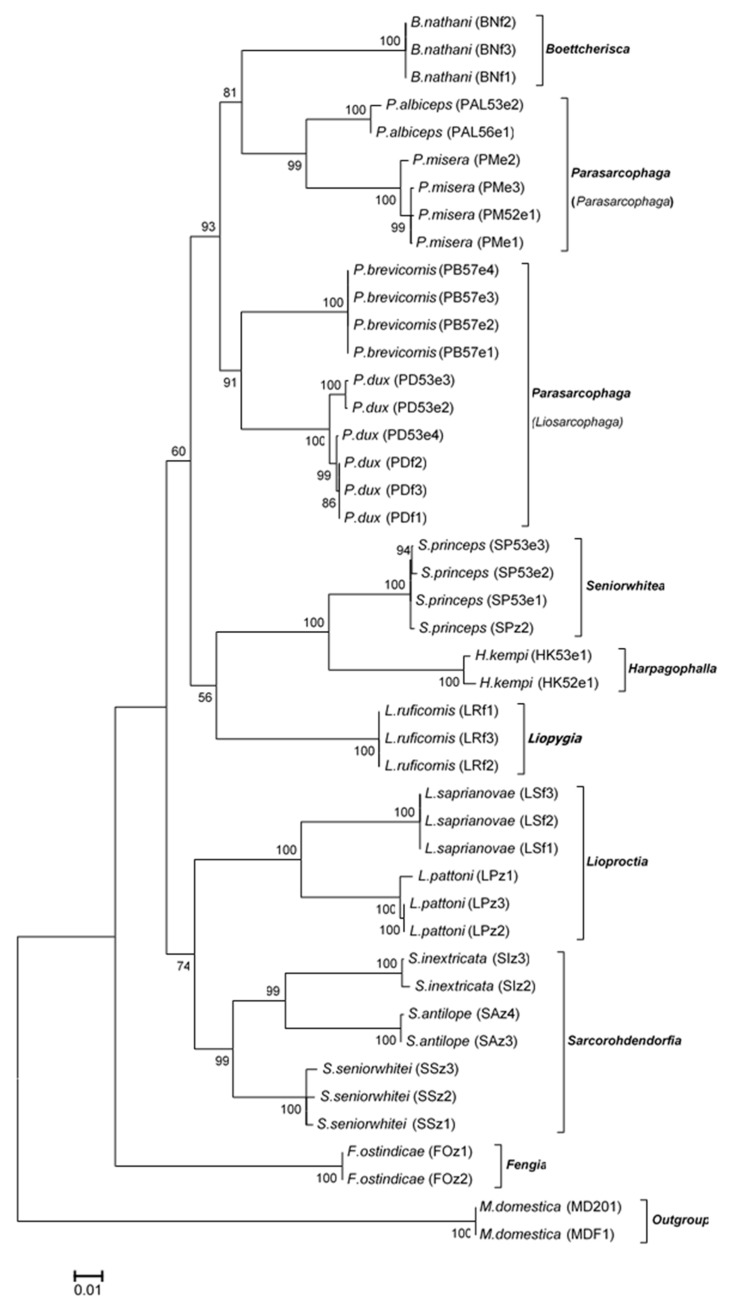
Maximum likelihood tree based on the combined *COI*-*COII* sequences of 14 Thai sarcophagid species. Species name, voucher code, and accession number are given in the specimen label. Bootstrap values are shown at each node. *M domestica* was taken as the outgroup. Evolutionary distance divergence scale bar is 0.01.

**Figure 3:**
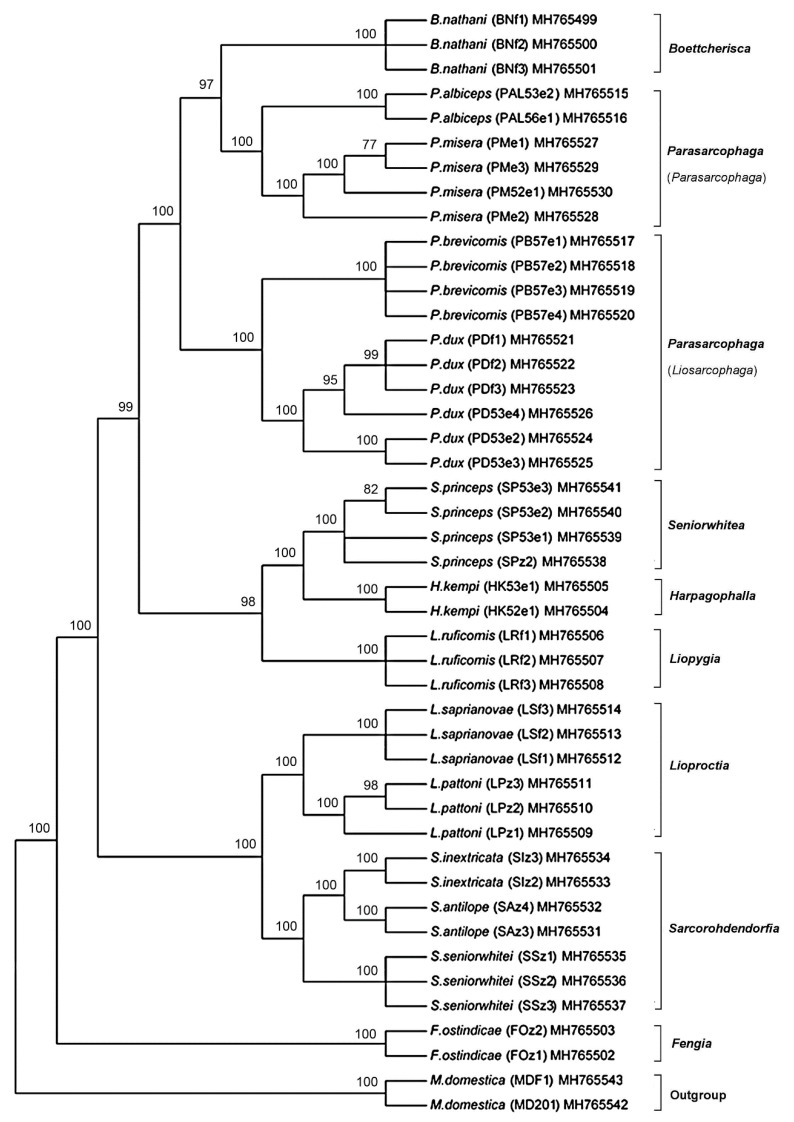
Bayesian inference tree based on the combined *COI*-*COII* sequences of 14 Thai sarcophagid species. Posterior probabilities are shown at each node. Species name, voucher code, and accession number are given in the specimen label. *M*. *domestica* was taken as the outgroup.

The ML and BI analyses based on the *COI*-*COII* sequences of the Thai flesh flies yielded similar tree topologies (Figure 2 and Figure 3). Both ML and BI trees clearly separated the flesh flies from the house fly outgroup. At the species level, the 14 flesh fly species formed their own monophyletic clusters with very strong supportive values. Two distinct clades of the Thai flesh flies were constructed. A major clade consisted of fly species in genera *Boettcherisca*, *Parasarcophaga*, *Seniorwhitea*, *Harpagophalla*, and *Liopygia*, while a minor one comprised those in genera *Lioproctia*, *Sarcorohdendorfia*, and *Fengia*. Within the genus *Parasarcophaga*, subgenera *Parasarcophaga* and *Liosarcophaga* were independently formed. Genus *Boettcherisca* and subgenus *Parasarcophaga* were resolved as sister clades. *F. ostindicae* in the clade of genus *Fengia* was distinct and placed far from other genera as an ancestor of all tested sarcophagids.

## 4. Discussion

Because of the high similarity in external appearance of sarcophagids, the morphology-based identification, therefore, required expert entomologists. In Thailand, the male genitalia are commonly used to define adults up to the species level [11]. Recently, wing morphometric analysis has been applied for species and sex discrimination of flesh flies in Thailand [39]. Nevertheless, the wing morphometry is feasible exclusively for the adult and sometimes impractical for incomplete specimens. To date, DNA-based identification has been widely used as an alternative method to solve the issues surrounding correct species identification based solely on morphological identification of sarcophagids. So far, only two genetic studies of forensically relevant flesh flies have been reported from Thailand. The first study used the short *COI* sequences (351 bp) to verify maggot species using PCR-RFLP [29]. Subsequently, the partial sequences of *COI* (648 bp) and *28S rRNA* (~1 kb) were utilized to discriminate 13 forensically important fly species (nine species of blow flies and four species of flesh flies), revealing higher potential of *COI* in species differentiation than *28S rRNA* [30]. In this study, the consecutive sequences of almost complete *COI-COII* genes (~2200 bp) of Thai flesh flies covering 14 forensically relevant species were, first, generated to evaluate their marker efficiency in correct species identification. The updated local genetic database should be useful for further molecular identification in the country. In addition, the long *COI*-*COII* sequences of *B*. *nathani*, *F*. *ostindicae*, and *S*. *seniorwhitei* were deposited into the GenBank for the first time.

To utilize the DNA sequences for accurate species discrimination, it is known that the genetic distances are very important parameters [16,34,40]. The overlapping of divergence percentages between intraspecific distances and interspecific distances can cause vagueness in species-level resolution. In this study, the distance-based analysis showed that the genetic diversity of the long *COI-COII* sequences was robust enough for discriminating 14 species of Thai flesh flies as there was no overlapping between the intra- and interspecific distances. Similar results have also been previously reported in the sarcophagids identification based on complete *COI-COII* genes from Malaysia [21] and China [41]. Among the examined species, it was noted that *P. dux,* in this study, showed a high value of mean intraspecific variation (0.96%, *n* = 3), which was in accordance with that reported from Malaysia (0.83%, *n* = 3) [21] and China (0.7%, *n* = 6) [41]. It was likely that the genetic divergences of most flesh fly species retrieved from biodiversity-rich countries such as Thailand (in this study) and Malaysia [21], were relatively high when compared with those from China [41,42]. For the specimens from the laboratory colonies (*B. nathani*, *L. ruficornis*, *L. saprianovae*, and some of *P. dux*), although they were selected from different isolines, they all showed no genetic diversity. This should be taken into account to the extent that laboratory colonies would not be proper representative samples for population genetics studies. Multiple genes analysis with an increasing number of samples per species from various field locations are needed to investigate the genetic deviations of the sarcophagid taxa in biodiversity-rich areas. Furthermore, not only the forensically important flesh fly species, but also other species of unexpected flesh flies should be concerned as they might be found on the remains.

The *COI-COII* sequences of Thai flesh flies showed the *tRNA-leu* gene which was connected by an intergenic spacer. Contents and arrangements of nucleotides in the regions, were almost conserved for all species examined and did not affect the sequence analysis. These findings also concurred with previous flesh fly studies which reported the high nucleotide conservation in both *tRNA-leu* gene and short intergenic spacers in the *COI-COII* genes [21,41]. For *tRNA* genes in mitochondrial genome, their conserved properties had little influent impact on the phylogeny reconstruction investigated from a horse stomach bot fly and myiasis fly [43]. For the intergenic spacer of Thai flesh flies, very short lengths (6 to 7 bp depended on the individual) in the spacer attributed to the existence of 2 bp indels after the final alignments. The presence of indels has been similarly reported in the mitochondrial study of flesh flies from Malaysia, Indonesia, Japan [21]. (2 bp indels), China [41] (5 bp indels), and Iran (1 bp indels) [44]. The differences of indels detected in the spacer alignments varied depending on spacer length, number of samples, genetic variations in fly species involving the geographical diversity, etc. It is known that mitochondrial genes of animals generally contain very short spacers [45] and are thought not to have any functions [46]. For the sequence-based species identification such as RFLP, although little variations in the short spacers might not have more impacts on the analysis than intraspecific variation in the genes, its effect on the alteration of restriction patterns could not be ignored. Therefore, the careful interpretation should be taken into account when intergenic spacers are included as part of the polymorphism analyses.

Regarding identification success proposed by Meier et al. [34]. BM, BCM, and ASB criteria mainly relied on the sequence pairwise comparison. Therefore, this method can be influenced by many factors, for example, number of specimens, diversity, and relatedness of species and geographical scale of sampling [17,30,34,40]. Particularly in BCM and ASB criteria, genetic distances are required for calculating the threshold value, thus conspecific and congeneric sequences may act as biased data and influence the calculated values [34]. In this study, 100% correct identification was found under the BM and BCM criteria, while only 76.74% correct identification was obtained from the ASB criterion because of the presence of ambiguous identifications (23.25%). The ambiguous identification resulted from the queries that had only one conspecific match in the dataset (i.e., *F*. *ostindicae*, *H*. *kempi*, *P*. *albiceps*, *S*. *antilope*, and *S*. *inextricata*). 

The identification success of flesh fly sequences based on *COI* gene under the BM and BCM criteria has been formerly published [24]. The results showed that the correct identification obtained by the short *COI* sequences of 127 bp (80.7% to 82.5%) was lower than that of the standard *COI* (658 bp) or entire *COI* (1535 bp) (98.2% to 99.3%) regions. Comparing the identification success rates based on the *COI* gene by Jordaens et al. [24], our study using *COI-COII* sequences showed 100% correct identification in both BM and BCM criteria. This suggested that the identification success rate could be improved by using the longer length of combined *COI-COII* sequences in the analysis.

The ML and BI phylogenies which included the nucleotide substitution model (GTR+I+G) in the analyses, showed similar tree topologies with greatly supported monophyletic groupings for all Thai sarcophagids at the species level. Remarkably, *B*. *nathani* (genus *Boettcherisca*) was phylogenetically placed closely to *P*. *albiceps* and *P*. *misera* (subgenus *Parasarcophaga*) as sister clades, even though their male genitalia are distinctively different [11,31]. The close genetic relationship of the sister grouping between subgenus *Parasarcophaga* and genus *Boettcherisca* has also previously been phylogenetically constructed based on the complete *COI-COII* genes based on species in other countries [21,43]. However, the phylogenetic placement of the sarcophagids analyzed by different markers such as the *period* nuclear gene [13] were different. For a better understanding of genetic variation within the flesh fly populations, further molecular research is required using other combined multiple genes [25] from more field samples captured in different geographical locations. 

In the tree-based analysis, Thai sarcophagids were all assigned to their respective species, corresponding well with their morphological identification by male genitalia [11]. Furthermore, the congruent correlations between setae features on the scutum (postsutural *dc*) and *COI-COII* based classification were also observed. The flesh flies that phylogenetically cluster in the major clade (genera *Boettcherisca*, *Parasarcophaga*, *Liopygia*, *Harpagophalla*, and *Seniorwhitea*) possessed the postsutural *dc 5* trait, while the remaining flies in the minor clade (genera: *Lioproctia*, *Sarcorohdendorfia,* and *Fengia*) showed postsutural *dc 4* type. The traits characterized in this study could be one of the sharing features among the genera based on genetically inherited traits from the same ancestor. The relationship between *COI* sequence variations and morphological features at terminalia has never been reported among the intraspecies of *P*. *dux* in Taiwan [47]. However, the impacts of sequence diversity on morphological variation are still controversial in scientific communities [48,49]. 

## 5. Conclusions

The combined *COI-COII* genes are potential genetic markers that can be used in combination with morphology-based tools for accurate species identification of Thai sarcophagids. With sufficient divergences, the genes showed a very high percentage of successful species identification. The achieved sequences covering 14 forensically important flesh fly species including the newly submitted sequences of *B*. *nathani*, *F*. *ostindicae* and *S*. *seniorwhitei*, can serve as a reliable reference database for further forensic entomological research not only in Thailand but also in the areas where those species are present. Although this study consisted of many flesh fly species of forensic importance of Thailand, only a few specimens of each species were used for analysis. Therefore, more specimens of individual species, as well as other species of forensically important flesh flies from several parts of Thailand, should be used to develope a comprehensive reference database and provide additional data on their genetic diversity.

## Figures and Tables

**Table 1:** Locality and reference data of specimens used in this study.

Species	Voucher Number	Locality	GPS Reference and Altitude (m)	Accession Number
*B. nathani*	BNf1 BNf2 BNf3			MH765499 MH765500 MH765501
		Laboratory colonies, Chiang Mai	-	
	
*F. ostindicae*	FOz1 FOz2	Hang Chat, Doi Khuntan, Lampang	18°23′30.90″ N 99°12′47.13″ E 531 m	MH765502 MH765503
*H. kempi*	HK52e1	Khon Kaen University, Khon Kaen	16°6.14′28″ N 102°1.49′19″ E	MH765504
	HK53e1	Warin Chamrap, Ubon Ratchathani University, Ubon Ratchathani	15°07′1.45″ N 104°54′8.41″ E	MH765505
*L. ruficornis*	LRf1			MH765506
	LRf2	Laboratory colonies, Chiang Mai	-	MH765507
	LRf3			MH765508
*L. pattoni*	LPz1	Doi Saket, Huai Hongkhrai Royal, Chiang Mai	18°53′57.54″ N 99°13′3.79″ E 487 m	MH765509
	LPz2	Hang Dong, Ban Pong, Chiang Mai	18°46′53.47″ N 98°51′18.86″ E 512 m	MH765510
	LPz3			MH765511
*L. saprianovae*	LSf1			MH765512
	LSf2	Laboratory colonies, Chiang Mai	-	MH765513
	LSf3			MH765514
*P. albiceps*	PAL53e2	Warin Chamrap, Ubon Ratchathani University, Ubon Ratchathani	15°07′1.45″ N 104°54′8.41″ E	MH765515
	PAL56e1	Thale Ban National Park, Satun	6°42′41.69″ N 100°10′11.22″ E 100 m	MH765516
*P. brevicornis*	PB57e1 PB57e2 PB57e3 PB57e4	Faculty of Veterinary Medicine, Prince of Songkla University, Hatyai Campus, Songkla	7°0′7.97″ N 100°30′4.32″ N 22 m	MH765517 MH765518 MH765519 MH765520
*P. dux*	PDf1			MH765521
	PDf2	Laboratory colonies, Chiang Mai	-	MH765522
	PDf3			MH765523
	PD53e2 PD53e3 PD53e4	Warin Chamrap, Ubon Ratchathani University, Ubon Ratchathani	15°07′45.1″ N 104°54′41.8″ E	MH765524 MH765525 MH765526
*P. misera*	PMe1 PMe2 PMe3	Suanpa Kaokrayang, Phitsanulok	16°50′46″ N 100°44′52″ E 179.5 m	MH765527 MH765528 MH765529
	PM52e1	Khon Kaen University, Khon Kaen	16°28′14.6″ N 102°49′49.1″ E	MH765530
*S. antilope*	SAz3 SAz4	Doi Saket, Doi Nang kaew, Chiang Mai	19°03′53″ N 99°22′34″ E, 974 m	MH765531 MH765532
*S. inextricata*	SIz2 SIz3	Doi Saket, Huai Hongkhrai Royal, Chiang Mai	18°53′57.54″ N 99°13′3.79″ E, 487 m	MH765533 MH765534
*S. seniorwhitei*	SSz1	Hang Chat, Doi Khuntan, Lampang	18°23′30.90″ N 90°12′47.13″ E, 531 m	MH765535
	SSz2 SSz3	Doi Saket, Doi Nang kaew, Chiang Mai	19°03′53″ N 99°22′34″ E, 974 m	MH765536 MH765537
*S. princeps*	SPz2	Doi Saket, Huai Hongkhrai Royal, Chiang Mai	18°53′57.54″ N 99°13′3.79″ E, 487 m	MH765538
	SP53e1 SP53e2 SP53e3	Warin Chamrap, Ubon Ratchathani University, Ubon Ratchathani	15°07′45.1″ N 104°54′41.8″ E	MH765539 MH765540 MH765541
*M. domestica*	MDF1	Laboratory colonies, Chiang Mai	-	MH765542
	MD201			MH765543

**Table 2:** The alignment of 7 nucleotide positions within a spacer region of 14 Thai flesh fly species in this study.

Species *	Spacer Region (Alignment Position)						
	1562	1563	1564	1565	1566	1567	1568
*B. nathani*	C	-	A	-	C	T	A
*P. albiceps*	T	-	A	A	C	T	A
*P. misera*	T	-	A	A	C	T	A
*P. brevicornis*	T	-	A	A	C	T	T
*P. dux*	T	T	A	A	C	T	T
*H. kempi*	T	T	A	A	T	A	A
*S. princeps*	A	T	A	A	T	A	A
*L. ruficornis*	C	T	C	A	C	T	A
*L. pattoni*	-	-	C	A	C	T	A
*L. saprianovae*	-	-	C	A	C	T	A
*S. antilope*	T	-	C	A	C	T	A
*S. inextricata*	T	-	T	A	T	T	A
*S. seniorwhitei*	T	-	T	A	T	T	A
*F. ostindicae*	A	C	C	A	C	T	A

* Each color indicates a different genus.

**Table 3:** Variable positions in the tRNA-leucine gene alignment of 14 Thai flesh flies in this study.

Species	Variable Position in tRNA-Leu Gene			
	1502	1510	1531	1534
*B. nathani*	A	T	C	T
*F. ostindicae*	*	*	T	*
*H. kempi*	*	*	*	*
*L. ruficornis*	*	*	*	*
*L. pattoni*	*	*	*	*
*L. saprianovae*	*	*	*	*
*P. albiceps*	*	*	*	*
*P. brevicornis*	*	*	*	*
*P. dux*	*	*	*	*
*P. misera*	*	*	*	*
*S. antilope*	G	C	*	G
*S. inextricata*	G	*	*	*
*S. seniorwhitei*	*	*	*	*
*S. princeps*	*	*	*	*

* The asterisk symbol indicates the same nucleotide at the equivalent position of *B*. *nathani*. Different bases in the alignment are in bold.

**Table 4:** Percentage of mean intraspecific variation and interspecific variation based on the *COI*-*COII* sequences in this study.

No.	Species	*N*	Mean Intraspecific Variation (%)	1	2	3	4	5	6	7	8	9	10	11	12	13	14
1	*B*. *nathani*	3	0.00	-													
2	*F*. *ostindicae*	2	0.00	11.87													
3	*H*. *kempi*	2	0.64	10.85	12.31												
4	*L*. *ruficornis*	3	0.00	9.97	11.15	9.93											
5	*L*. *pattoni*	3	0.59	10.84	11.78	10.87	9.85										
6	*L*. *saprianovae*	3	0.00	11.17	11.73	11.06	10.40	6.77									
7	*P*. *albiceps*	2	0.36	8.04	10.75	10.34	8.18	10.29	10.69								
8	*P*. *brevicornis*	4	0.00	8.05	10.35	9.92	8.15	9.16	10.12	7.12							
9	*P*. *dux*	6	0.96	8.10	9.85	9.48	8.46	9.94	10.01	6.55	5.72						
10	*P*. *misera*	4	0.73	8.05	11.12	10.82	9.11	11.42	10.71	5.22	7.78	7.49					
11	*S*. *antilope*	2	0.09	10.66	11.31	11.45	10.33	10.02	10.54	10.57	9.11	9.47	10.98				
12	*S*. *inextricata*	2	0.32	10.51	11.19	10.76	1073	10.24	10.06	10.45	9.22	9.11	10.68	6.68			
13	*S*. *seniorwhitei*	3	0.64	9.27	9.38	9.36	9.44	8.36	8.22	8.46	7.53	7.75	8.76	6.89	6.50		
14	*S*. *princeps*	4	0.36	9.88	11.98	6.62	8.78	10.51	10.71	8.94	8.74	8.45	10.11	10.12	10.84	8.90	-

**Table 5:** Identification success based on Best Match (BM), Best Close Match (BCM) and All Species Barcodes (ASB) criteria.

Criteria	*COI*-*COII*
No. of sequences	43
No. of sequences with at least 1 matching conspecific sequence	43
No. of sequences with closet match at 0% difference	20
No. of allospecific matches at 0% difference	0
Best Match (BM)	
Correct identifications Ambiguous identifications Incorrect identifications	100% (43) 0.00% (0) 0.00% (0)
Calculated threshold for Best Match and All Species Barcodes	0.94%
Best Close Match (BCM)	
Correct identifications Ambiguous identifications Incorrect identifications No match closer than the calculated threshold	100% (43) 0.00% (0) 0.00% (0) 0.00% (0)
All Species Barcodes (ASB)	
Correct identifications Ambiguous identifications Incorrect identifications No match closer than the calculated threshold	76.74% (33) 23.25% (10) 0.00% (0) 0.00% (0)
